# Incorporating TiO_2_ nanotubes with a peptide of D-amino K122-4 (D) for enhanced mechanical and photocatalytic properties

**DOI:** 10.1038/srep22247

**Published:** 2016-02-26

**Authors:** L. Q. Guo, Y. W. Hu, B. Yu, E. Davis, R. Irvin, X. G. Yan, D. Y. Li

**Affiliations:** 1 Department of Chemical and Materials Engineering, University of Alberta, Edmonton, AB T6G 2V4, Canada; 2 Department of Medical Microbiology and Immunology, University of Alberta, Edmonton, Alberta, Canada; 3 School of Mechanical Engineering, Taiyuan University of Science and Technology, Taiyuan, Shanxi, 030024, People’s Republic of China

## Abstract

Titanium dioxide (TiO_2_) nanotubes are promising for a wide variety of potential applications in energy, biomedical and environmental sectors. However, their low mechanical strength and wide band gap limit their widespread technological use. This article reports our recent efforts to increase the mechanical strength of TiO_2_ nanotubes with lowered band gap by immobilizing a peptide of D-amino K122-4 (D) onto the nanotubes. Topographies and chemical compositions of the peptide-coated and uncoated TiO_2_ nanotubular arrays were characterized by scanning electron microscopy and X-ray photoelectron spectroscopy (XPS). Properties of the peptide-coated and uncoated TiO_2_ nanotubular arrays, including hardness, elastic modulus, electron work function and photocurrent, were evaluated using micromechanical probe, Kelvin Probe and electrochemical system. Effect of the peptide on surface conductivity was also investigated through current mapping and I–V curve analysis with conductive atomic force microscopy. It is demonstrated that the peptide coating simultaneously enhances the mechanical strength, photocatalytic and electrical properties of TiO_2_ nanotubes.

Titanium dioxide nanotubes (TNTs) have demonstrated great potential for applications in various technical fields, e.g., environmental purification, photocatalysts, gas and humidity sensor and solar energy conversion, etc.^1^,^2^,^3^,^4^,^5^,^6^,^7^,^8^. However, low mechanical strength and brittleness as well as wide band gap impair their feasibility for widespread technological use. Many efforts have been made to improve the photocatalytic activity of TiO_2_ nanotubes through element doping and utilizing different fabrication methods^8^,^9^,^10^,^11^,^12^,^13^,^14^,^15^,^16^. However, the work on the mechanical behavior of TiO_2_ nanotubes is rather limited, though some relevant studies have been reported in the literature^17^,^18^,^19^,^20^. No effective systems and methods have been developed to fabricate TiO_2_ nanotubes with simultaneously improved mechanical strength, photocatalytic activity and electrical properties.

Here, we demonstrate a new approach to simultaneously improve the mechanical strength, photocatalytic and electrical properties of TiO_2_ nanotubes by incorporating TiO_2_ nanotubes with a peptide of D-amino K122-4 (D)^21^,^22^, which was fabricated by solid-phase peptide synthesis and purified with reversed-phase high-performance liquid chromatography (HPLC). Previous work^23^,^24^ demonstrated that this peptide interacted with the stainless steel surface and affected its surface electron state and consequently the mechanical strength and other properties such as the antibacterial capability. It was observed that the peptide-modified 304 steel surfaces were considerably harder than untreated ones^23^. This synthesized peptide constitutes the self-organizing or folding PilA receptor binding domain (RBD)–(a synthetic peptide consisting of residues 128–144 from *Pseudomonas aeruginosa* with a formed disulfide bridge) and displayed a remarkably high apparent binding affinity for steel. The chiral form of amino acids (the D-enantiomeric form) has been used to overcome protease sensitivity of peptides such that they can be used for clinical applications. D-amino acid residues can be incorporated into the peptide at protease cleavage sites to prevent the recognition of the peptide by proteases.

Effects of the peptide immobilized on TiO_2_ nanotubular arrays on their properties were evaluated, including surface hardness, elastic modulus, electron work function (EWF), photocurrent and conductivity.

## Results and Discussion

### Surface characterization

**Figure 1:**
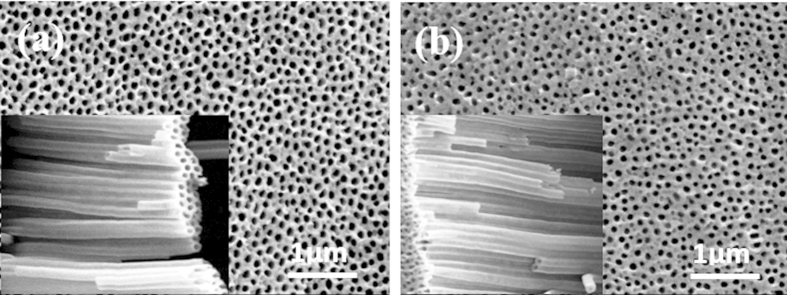
SEM images of uncoated (**a**) and peptide-coated (**b**) TiO_2_ nanotubular arrays (top view) and inserts show side views.

Figure 1 illustrates the top views and cross-sectional view of TiO_2_ nanotubular arrays before (Fig. 1a) and after (Fig. 1b) being coated with the peptide observed by SEM. The average outer diameter of the uncoated TiO_2_ nanotubes is about 100 nm, and the wall thickness is approximately 10 nm. The average outer diameter and wall thickness of the peptide-coated TiO_2_ nanotubes are similar to those of the uncoated ones but the inner diameter is slightly smaller than that of the uncoated nanotubes after the peptide was coated, indicating that the peptide coating is quite thin. These results were expected, as the peptide itself is only several Angstroms in size and previous work has shown that a concentration of 10 ug/mL of peptide saturates surfaces and analysis of coated surfaces by AUGER-SEM suggested a uniform distribution of the peptide on the surface^23^.

**Figure 2:**
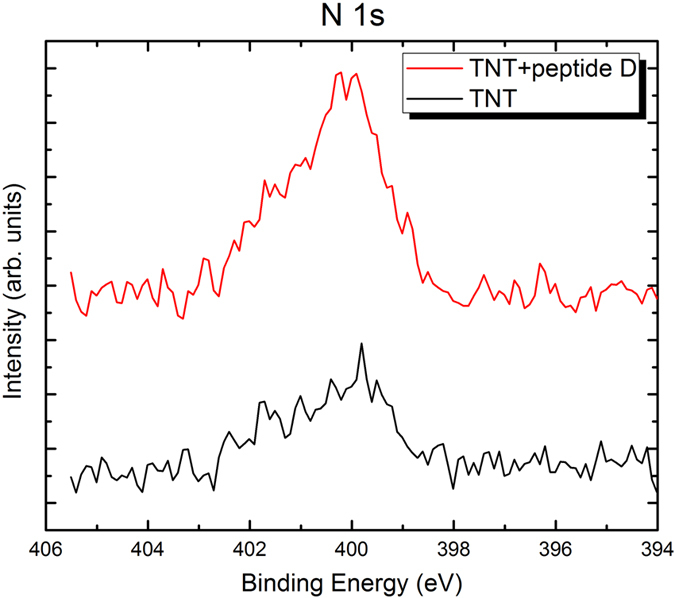
XPS spectra of N 1s of the uncoated (black color) and peptide-coated (red color) TiO_2_ nanotubes. The N signal of the peptide-coated sample is stronger than that of the uncoated one.

**Figure 3:**
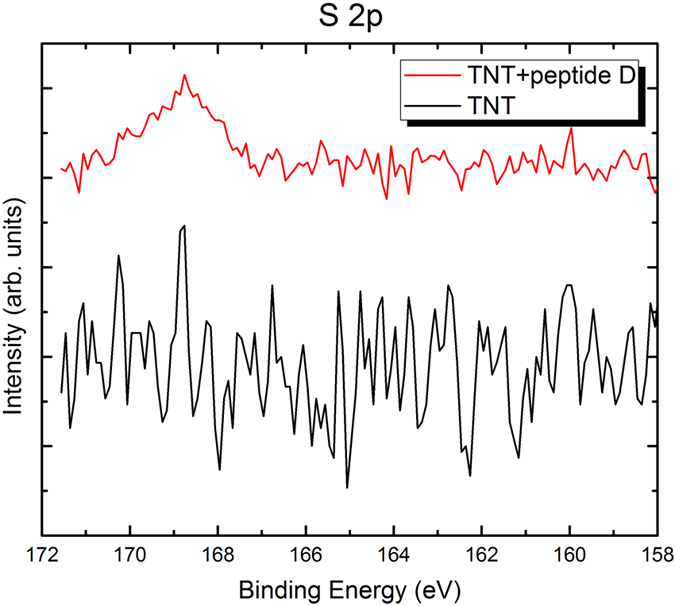
XPS spectra of S 2p of the uncoated (black color) and peptide-coated (red color) TiO_2_ nanotubes. The peptide-coated sample has a clear S peak, while the uncoated one does not show any meaningful S signal except noise.

**Figure 4:**
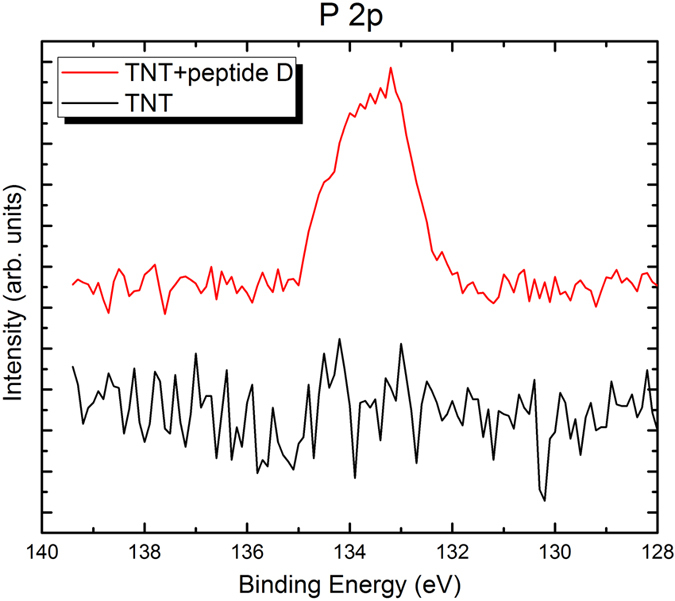
XPS spectra of P 2p of the uncoated (black color) and peptide-coated (red color) TiO_2_ nanotubes. The peptide-coated sample has a clear P peak, while the uncoated one does not show any meaningful P signal except noise.

XPS analysis was carried out to analyze changes in surface composition caused by the peptide coating. From obtained XPS spectra shown in Figs 2, 3, 4, clear nitrogen, sulfur and phosphorus were detected on the peptide-coated TiO_2_ nanotubular arrays, in comparison with the uncoated TiO_2_ nanotubular arrays. Quantitative XPS analysis showed 3.03% of N, 0% of S and P (i.e. no S and P), and 96.97% of Ti (Ti 2p spectrum is not shown) in the uncoated TiO_2_ nanotubular arrays. In comparison, the peptide-coated ones have higher concentrations of nitrogen, sulfur and phosphorus, which are 6.14% of N, 0.86% of S, 3.32% of P, respectively. Moreover, the atomic concentration ratio of nitrogen to titanium of the peptide-coated TiO_2_ nanotubular arrays (N/Ti = 1:10) is much higher than that of the uncoated ones (N/Ti = 1:33), meaning that the nitrogen content was significant increased as the peptide was coated. This confirms that the peptide was immobilized on the TiO_2_ nanotubes, since nitrogen is one of major elemental components of the peptides. Besides, the peptide has two cysteine amino acids which contain sulfur, forming a disulphide bridge, whereas the titanium should not contain sulfur, suggesting that the detected sulfur comes from the peptide immobilized on the surface. Although the peptide does not contain any phosphorus, phosphate-buffered saline (PBS) was used to dissolve the peptide which may explain why the element P was present on the peptide-coated nanotubes. Thus, we conclude that the peptide was indeed immobilized on the TiO_2_ nanotubes. It needs to be indicated that, XPS spectra of other elements such as oxygen, titanium and carbon in the peptide-coated and non-coated TiO_2_ nanotubes were also obtained. Since little changes are observed in terms of the intensity distribution and corresponding electronic state (their XPS spectra are therefore not provided here), the peptide should be simply bound to the surface of nanotubes.

### Hardness and Young’s modulus

**Figure 5:**
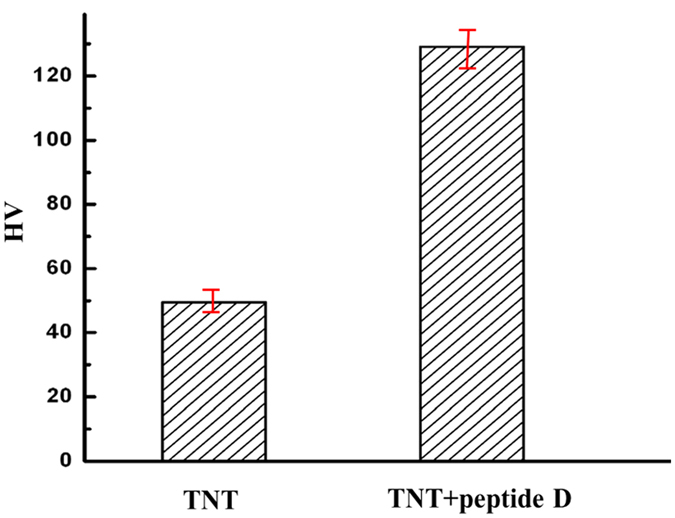
Hardness values of TiO_2_ nanotubes without (left) and with peptide (right), averaged over five measurements. The hardness of peptide-coated nanotubular arrays is about 2.6 times as large as that uncoated TiO_2_ nanotubular arrays.

**Figure 6:**
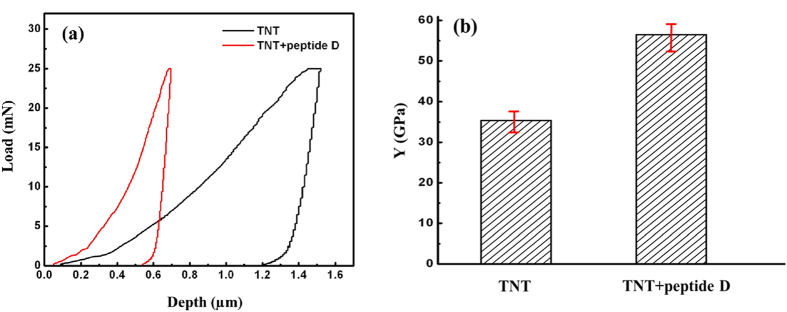
(**a**) Load-depth curves, and (**b**) Young’s moduli of TiO_2_ nanotubes without peptide (TNT) and with peptide (TNT + peptide D), averaged over five measurements. Young’s modulus of the peptide-coated nanotubular arrays is 1.63 times as large as that of the uncoated ones.

A micromechanical probe was used to evaluate effects of the coated peptide on hardness and Young’s modulus of the nanotubular arrays under a small load of 25 mN. Values of hardness and Young’s modulus were obtained from the load-indentation depth curve, automatically calculated by the micro-indentation system with WIN-HCU® software, which used Oliver-Pharr model to analyze the obtained load-depth curve. Results of the experiment demonstrated that hardness of the peptide-coated nanotubular arrays was 2.6 times as large as that uncoated TiO_2_ nanotubular arrays, as shown in Fig. 5. It is obvious that the peptide effectively strengthened the TiO_2_ nanotubular arrays. Figure 6 illustrates load-depth curves of peptide-coated nanotubes and uncoated TiO_2_ nanotubes, from which their elastic moduli were determined. Young’s modulus of the peptide-coated nanotubular arrays was 1.63 times as large as that of the uncoated ones. TiO_2_ nanotubes are brittle in nature and have low resistance to external force. When subjected to indentation, the nanotubes collapse and cause permanent damage accompanied with a larger irreversible indentation depth, leading to not only low apparent hardness but also low apparent elastic modulus. When the nanotubes were coated with the peptide, this polymeric substance played a role as a binder, which strengthened the brittle nanotubes with elevated toughness. As evidenced by the indentation tests, the peptide coating clearly improved the mechanical behavior of the TiO_2_ nanotubular arrays and corresponding resistance to indentation.

### Effects of the peptide on EWF and photocurrent of TiO_2_ nanotubular arrays

**Figure 7:**
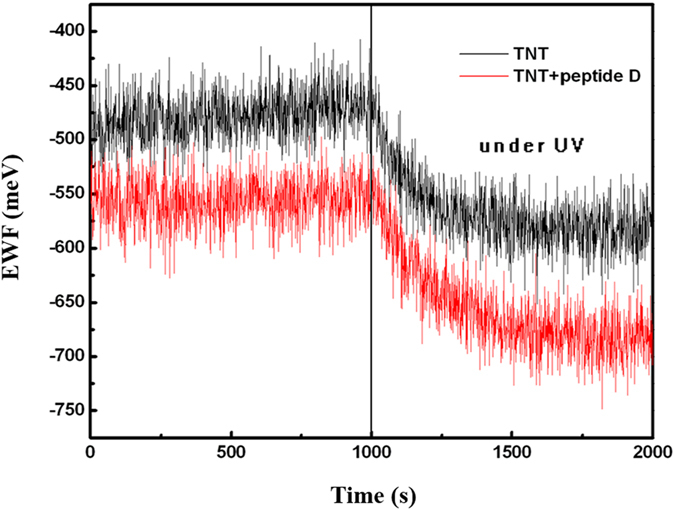
Changes in the electron work function of peptide-coated (red color) and uncoated (black color) TiO_2_ nanotubular arrays under illumination of UV-vis light with time. EWF of the peptide-coated TiO_2_ nanotubues is lower than that of the uncoated nanotubes, indicating that the former are more electrically active than the uncoated ones. When the illumination was turned on, EWF of the peptide-coated nanotubes decreased more than the uncoated ones, implying that the peptide enhanced the sensitivity of the nanotubes to illumination and thus facilitated the photon-induced electron excitation.

Changes in the electron work function (EWF) of peptide-coated and uncoated TiO_2_ nanotubular arrays under illumination were monitored (Fig. 7). The presented are values relative to that of Au as a reference (5.1 meV) rather than absolute EWF values. The work function is the minimum energy required to move electrons from inside a solid at Fermi level to its surface without kinetic energy. This parameter is a measure of the electron activity. As illustrated in Fig. 7, the EWF of the peptide-coated TiO_2_ nanotubes is lower than that of the uncoated nanotubes, indicating that the coated nanotubes are more electrically active than the uncoated ones. As shown, when the illumination was turned on, EWFs of both samples decreased and the peptide-coated nanotubes decreased more than the uncoated ones. This suggests that the peptide enhanced the sensitivity of the nanotubes and facilitated the photon-induced electron excitation. The excitation of electrons in TiO_2_ nanotubes is an electron-hole separation process; facilitation of the electron-hole separation by the peptide benefits the photocatalytic capability of the TiO_2_ nanotubes.

**Figure 8:**
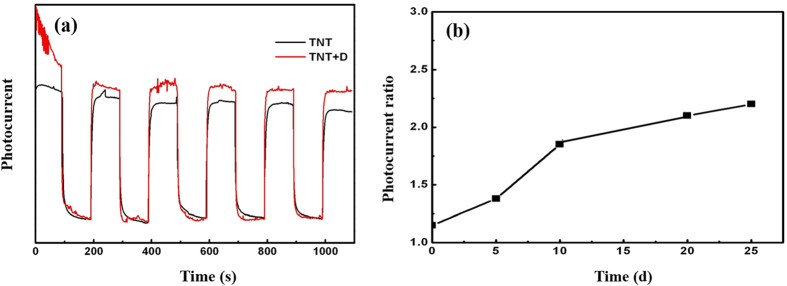
(**a**) Photocurrent responses of TiO_2_ nanotube samples with and without the peptide to pulsed irradiation of UV-vis light; TNTs with peptide D have larger photocurrents than TNTs without peptide; and the photocurrent ratio of TNT with peptide D to TNT is about 1.15. (**b**) The photocurrent responses of the samples were measured again after 5, 10, 20 and 25 days, respectively. The photocurrent ratio of TNT with peptide D to TNT increased with time. The increase in the ratio was rapid initially and slowed down, which should approach a saturated value as the time is prolonged. Within the period under study, the ratio reached a value of 2.2 at day 25.

The beneficial effect of the peptide on the efficiency of photon-induced electron excitation is further confirmed by observed difference in photocurrent between the peptide-coated and unmodified TiO_2_ nanotubes. We measured their photocurrents under pulsed irradiation of UV-vis light. Induced photocurrents of both peptide-coated and uncoated TiO_2_ nanotubes well respond to the pulse UV-vis light. As shown in Fig. 8, the peptide-coated TiO_2_ nanotubes exhibited stronger photocurrent than the uncoated nanotubes, which is consistent with the result of EWF measurement. The peak photocurrent of the peptide-coated TiO_2_ nanotubes is about 15% higher than that of the uncoated ones, resulting from easier photo-induced electron excitation. In order to evaluate the stability of the photocurrent response, we performed the same tests for the samples after 5, 10, 20 and 25 days, respectively. It is interesting to observe that the ratio of photocurrent of the peptide-coated TiO_2_ nanotubes to that of uncoated ones increased with time from 1.15:1 to 2.2:1. The increase in the ratio is rapid initially and slows down, which should approach a saturated value as the time is prolonged. It is unclear why photocurrent ratio increased with time. The peptide is very stable. D-amino acid residue containing peptides are observed naturally in a variety of bacterial cell walls, and an interesting study looked at the half-life of those peptides in ocean sediment and found that it was extremely long, in the order of 100,000 plus years in a variety of ocean sediments where the dead bacteria had sedimented. Thus, we suspect the increased current flow may reflect a conformational shift of the peptide over time of exposure to the light source. Certain 3-dimensional shapes of the peptide when bound to the substrate are present and that accumulated energy state of the metal surface due to light exposure will begin to accumulate a structure that is less abundant in lower energy states. The higher energy state of the peptide conformation then likely has a higher efficiency of electron release which is then observed as an increase in current flow but this should stabilize at a certain stage. Further studies are needed in order to understand the observed phenomenon.

### Current measurements and I-V characteristics

**Figure 9:**
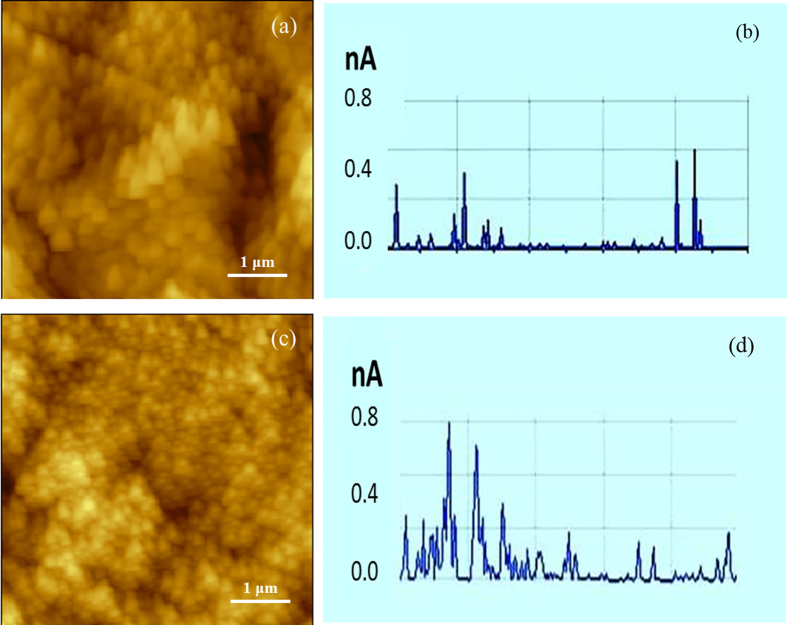
AFM topography image (**a,c**) and corresponding line-scanned current profiles (**b,d**) of the uncoated (**a,b**) and peptide-coated (**c,d**) TiO_2_ nanotubular arrays. A cut-off is set to only show major current signals. Unit of the current profile: nA. The peptide-coated sample is more electrically conductive with larger current.

To further investigate and understand the effects of the peptide on TiO_2_ nanotubes, we measured the electrical conductance of the coated and uncoated TiO_2_ nanotubes using a multimode AFM. Figure 9 illustrates topographical images and representative line-scanned current profiles of the peptide-coated and uncoated TiO_2_ nanotubular arrays, which show that the peptide-coated TiO_2_ nanotubes have a larger current than the untreated ones at the same applied tip bias (Fig. 9(b,d)), i.e. the peptide-coated nanotubes are more electrically conductive than the uncoated ones. The average current of the peptide-coated nanotubes is about 0.3 nA, while that of the uncoated nanotubes is about 0.1 nA.

**Figure 10:**
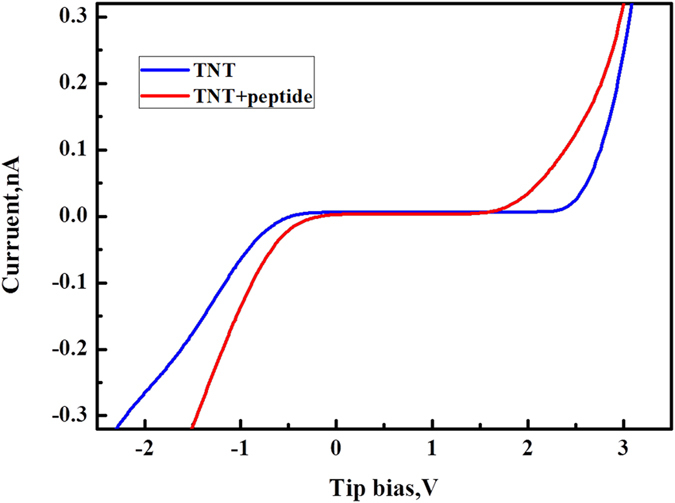
Typical I-V curves obtained from uncoated (blue color) and peptide-coated (red color) TiO_2_ nanotubes. The peptide-coated sample shows larger current than the uncoated one at the same applied bias. The zero-current region corresponds to the energy band gap. The width of the zero-current region of the peptide-coated sample is about 1.8 eV, much smaller than that of the uncoated nanotubes which is about 2.7 eV.

To better understand the electric characteristics of the peptide-coated and uncoated nanotubes, their I-V curves were measured and are illustrated in Fig. 10. The I-V curves demonstrate that the peptide-coated nanotubes have a higher conductivity than the uncoated ones, which is consistent with the current profiles shown in Fig. 9(b,d). The width of the zero-current region on the I-V curve of the peptide-coated nanotubes is about 1.8 eV, which is markedly smaller than that of the uncoated nanotubes (about 2.7 eV), as shown in Fig. 10. The zero-current region corresponds to the energy band gap, which is in good agreement with values of TiO_2_ nanotubes obtained using other techniques such as photoemission, UV absorption spectroscopy and calculation using the ab initio method^25^,^26^,^27^. The band gap energy (zero-current region width) of the uncoated TiO_2_ nanotubes extracted from I-V curve is consistent with values reported in the literature. Thus, the reduction of band gap energy of TiO_2_ nanotubes down to 1.8 eV by the peptide coating can be confirmed. The lower band gap of the peptide-coated nanotubes may explain why the paptide-coated nanotubes are more electrically conductive (see Fig. 9) and easier to be exited, as shown by the larger decrease in electron work function of the peptide-coated nanotubes under illumination (Fig. 7). As for the mechanism responsible for the effect of the peptide on band gap reduction, this could be related to fact that peptides are enriched in charged amino acids^28^,^29^,^30^. However, further studies are needed.

### Remark comments

Our original expectation is to mechanically strengthen the TiO_2_ nanotubes using the transparent peptide without deteriorating their photocatalytic properties. It is surprising to observe that the peptide does not only mechanically strengthen the TiO_2_ nanotubes but also enhances their photocatalytical activity with reduced band gap and elevated surface conductivity. The reduced band gap promotes the electron-hole separation and the higher surface conductivity facilitates charge transfer, thus reducing the chance for electron-hole recombination, leading to enhanced photocatalytic activity, which would benefit other related properties such as antimicrobial and pollutant degradation capabilities.

## Conclusions

TiO_2_ nanotubes are not mechanically strong (brittle) for feasible applications. In order to improve their mechanical strength, a transparent peptide of D-amino K122-4 (D) was coated on fabricated TiO_2_ nanotubular arrays, which effectively strengthen TiO_2_ nanotubes with considerably increased hardness, elastic modulus and resistance to indentation. Unexpectedly, it was observed that the peptide-coated nanotubes exhibited markedly increased photocatalytic activity with higher photocurrent, lower electron work function, higher surface conductivity, and smaller band gap. Such improvements would render the peptide coating as an effective modifier to TiO_2_ nanotubular arrays not only for enhanced mechanical durability but also improved photocatalytic and electrical properties.

## Methods

### TiO_2_ nanotubes fabrication

Highly ordered TiO_2_ nanotubular arrays were fabricated through potentiostatic anodization in a conventional two-electrode electrochemical cell at room temperature. A 0.2 mm thick titanium foil of 20 mm × 20 mm was used as the anode, and a platinum foil of 10 mm × 10 mm served as the cathode. The distance between the anode and the cathode was about 20 mm. The anodization was carried out under 50 V using a DC power supply for 1 h. Prior to anodization, titanium foils were soaked in a solution HF, HNO_3_, and H_2_O (1:3:10) for 40 s to remove the oxide layer on the surface. The electrolyte was composed of 0.25 wt% NH_4_F, 2.0 vol% H_2_O, and ethylene glycol. The as-prepared TiO_2_ nanotubular arrays were finally annealed at 450 °C for 2 h in a tube furnace with a heating/cooling rate of 1 °C·min^−1^ in the air atmosphere.

### Immobilization of peptide on TiO_2_ nanotubes

The TiO_2_ nanotubular arrays were coated with the D-amino K122-4 (D) peptide. The D-peptide ACTSNADNKYLPKTCQT-amide peptide, corresponding to amino acids 128-144 of the PilA receptor binding domain of *Pseudomonas aeruginosa* strain K122-4 was synthesized, by solid-phase peptide synthesis and purified with reversed-phase high-performance liquid chromatography (HPLC) as previously described by Wong *et al.*^21^,^22^. The disulphide bridge form of the peptide used in this study was generated by air-oxidization^23^. Purity of the peptide was verified by HPLC analysis and mass spectroscopy. Purity of the peptide was greater than 95%. The peptide was synthesized by the Peptide and Protein Chemistry Core Facility of the University of Colorado Health Sciences Center and reconstituted in phosphate buffered saline (PBS), pH 7.4. During the peptide coating process, the TiO_2_ nanotubular arrays were immersed in 95% EtOH for 15 min at room temperature with gentle agitation, the samples were then cleaned with acetone and gently shaken for 1 min, and finally washed with water and air dried. The cleaned samples were immersed into a 10 ug·mL^−1^ peptide MeOH solution for 1 h at room temperature with gentle agitation. After air drying, the samples were stored at room temperature for weeks. When necessary, the samples were rinsed with distilled water, or any concentration of MeOH or EtOH.

### Surface characterization

A JEOL JSM6301FXV scanning electron microscope (SEM) with a field emission electron source running a 5 KV was used for the microstructure characterization of the TiO_2_ nanotubular arrays with and without the peptide coating. Chemical state analysis of the samples was carried out with X-ray photoelectron spectroscopy (XPS) using a Kratos AXIS Ultra X-ray photoelectron spectrometer. A monochromatic Al source, operating at 210 W with a pass energy of 20 eV and a step of 0.1 eV, was utilized. All XPS spectra were obtained using the C1s line at 284.6 eV.

### Properties characterization

Microhardness of as-fabricated TiO_2_ nanotubular arrays with and without the coated peptide was determined using a micromechanical probe (Fischer Technology Ltd, Winsor, CT) under a small load of 25 mN. Each reported hardness value is an average of at least five measurements. Young’s modulus was determined from the load-unload curves recorded during the microindentation test.

Electron work function (EWF) of the samples was determined using a scanning Kelvin Probe (SKP 5050, KP Technology, UK). The suspension system produces oscillations with amplitudes in the range of 1–2 mm, permitting a standoff distance, which is regulated in the region 0.1–1.0 mm, ensuring that tip-to-sample contact does not occur.

Photocurrents of the samples were measured using a commercial electrochemical system. A standard three-electrode system with a platinum foil as the counter electrode and a saturated calomel electrode (SCE) as the reference electrode was used. The electrolyte was a 0.5 mol·L^−1^ Na_2_SO_4_ aqueous solution, and the potential used for the electrochemical analysis was set as 0.1 V vs SCE. A lamp power supply (LPS-220B, Photon Technology International, NJ, USA) was used as the light source for the photocurrent measurement.

The conductivities of TiO_2_ nanotubes with and without the coated peptide were measured using Agilent 5500 AFM (Agilent Technologies, USA) operated in the current sensing mode. In addition to topography and current maps, current-voltage (I-V) curves were acquired. Both current and I-V measurements were carried out in the ambient environment with its relative humidity around 25%.

## Additional Information

**How to cite this article**: Guo, L. Q. *et al.* Incorporating TiO_2_ nanotubes with a peptide of D-amino K122-4 (D) for enhanced mechanical and photocatalytic properties. *Sci. Rep.* **6**, 22247; doi: 10.1038/srep22247 (2016).
